# HIV/AIDS-associated beliefs and practices relating to diet and work in southeastern Uganda

**DOI:** 10.3329/jhpn.v28i1.4526

**Published:** 2010-02

**Authors:** Maction K. Komwa, Kathryn H. Jacobsen, Dawn C. Parker

**Affiliations:** ^1^ Department of Environmental Science and Policy; ^2^ Department of Global and Community Health; ^3^ Department of Computational Social Science, George Mason University, Fairfax, Virginia, USA

**Keywords:** Acquired immunodeficiency syndrome, Beliefs, Cross-sectional studies, Diet, HIV, Knowledge, attitudes, and practice, Nutrition, Uganda

## Abstract

To explore beliefs relating to diet, work, and HIV/AIDS among the Busoga of rural southeastern Uganda, a cross-sectional survey of 322 adults was conducted in 2007 in Mayuge district, Uganda. Of these adults, 56 were HIV-infected, 120 had a family member with HIV/AIDS, and 146 were in households without HIV-infected members. More than 74.2% of the adults knew someone with HIV/AIDS, and more than 90% correctly identified transmission modes and prevention methods of HIV. In total, 93.2% believed that a person with HIV should work fewer hours to conserve energy but all the three participant groups reported the same working hours. Also, 91.6% believed that a person with HIV infection should eat special nutritious foods, and the participants with HIV infection reported eating more fruits (p=0.020) and vegetables (p=0.012) than other participants. The participants expressed a consistent set of health beliefs about practices relating to HIV/AIDS.

## INTRODUCTION

HIV/AIDS is a major public-health problem in Uganda and throughout sub-Saharan Africa. Although the prevalence of HIV infection among adults aged 15–49 years in Uganda decreased from 18.3% in 1992 to 6.7% in 2005, acquired immunodeficiency syndrome (AIDS) continues to be the leading cause of death among adults in Uganda (1–4). The majority of deaths due to HIV/AIDS occur in the age-group that has the highest expectations for labour output and care-giving, and the loss of these household members has significant negative impact on the socioeconomic, nutritional and health status of affected households, their extended families, and their communities.

HIV/AIDS, work, and diet are all intricately related in HIV-affected households. In rural areas, reduction in labour-supply can lead to decreased agricultural productivity at the household level, which can, in turn, cause decreased nutritional status of household members and a concomitant reduction in health status and labour output (5). It is usually not possible for these households to purchase additional food due to loss of contributions to labour and income by both HIV-infected household members and their caregivers and because they have increased medical expenses that require out-of-pocket expenditure (6–9). Thus, although in some areas rural households with the capacity to engage in subsistence farming have greater food security and a more varied diet than their urban counterparts (10), any reduction in food production means that HIV-affected households are at an increased risk of hunger and extreme poverty (5).

These relationships among work, diet, and health status can theoretically lead to a downward spiral in which the poor health of one or more household members leads to reduced household work-output, leading to food shortages and nutritional deficits, which further decrease health status and labour productivity of the household. Due to these widely-recognized inter-relationships, government and non-governmental community organizations in Uganda have taken steps to try to interrupt the cycle of HIV/AIDS, hunger, and poverty. The Ugandan Government has issued nutritional guidelines for people with HIV, which suggest that people with HIV infection eat ‘special foods’, including various energy-giving foods (carbohydrates), body-building foods (proteins), protective foods (fruits and vegetables), and ample amounts of water (from sources including tea, soups, milk, and juices) (11, 12). This message is also promoted by local AIDS support groups, which provide food assistance to HIV-affected households, a programme that has been shown to allow persons with HIV infection to receive the essential nutrients they require (12, 13). Other community-specific recommendations for addressing concerns relating to HIV/AIDS are locally developed, based on the experiences and needs of each individual community.

The goal of this paper was to explore health knowledge, beliefs, and practices relating to HIV/AIDS, health status, labour, and diet in communities in rural southeastern Uganda using data from a cross-sectional survey conducted in 2007. The 2007 survey was designed to further investigate responses from 34 local community leaders who participated in focus groups in 2006. The groups comprised government officials, health officials, and employees of community-based non-governmental organizations; about 45 members of the community and AIDS support groups also participated in discussions. Two commonly-mentioned methods for improving the health status and extending healthy life for people with HIV infection emerged from these meetings. First, the interviewees reported that HIV/AIDS counsellors and healthcare officials advised people with HIV infection to limit their working hours and the intensity of their work, even if they felt healthy enough to work longer days at more labour-intensive jobs, to save energy and, thus, extend both years of life and healthy years of life. Second, they reported that people with HIV infection should eat ‘special foods’ from the time they are diagnosed through the end of their lives. This paper examined the extent to which these beliefs were held and acted upon in Mayuge district, Uganda, which is located along the shore of the Lake Victoria about 120 km east of Kampala, the national capital, and is heavily reliant on subsistence agriculture and fishing (14–16).

## MATERIALS AND METHODS

### Eligible population

The district officials provided the names of the chairpersons of all the subcounties and villages within Mayuge district, along with statistics on district population from the 2002/2003 National Household Survey (17). The local chairpersons in these communities act as government facilitators, mobilize community activities within their villages, and maintain a village census book that contains the names of all the households and individuals living within the areas under their authority. This ledger is used for identifying households eligible for government services, such as free food distribution, and for planning and coordinating village activities. The local chairpersons provided a list of all the households in their villages to the research team, along with general information about socioeconomic and demographic characteristics of each community, prevalent diseases, access to and use of health services, including antiretroviral (ARV) drugs for persons with AIDS, availability of safe drinking-water, access to schools, condition of roads, and the location of the nearest towns and shopping centres.

### Sampling

Three methods were used for recruiting 246 households for participation from an estimated 965 total households (for a yield of 25.5% of all households). The number of recruited households was based on estimations of sample size conducted using the StatCalc software (Epi Info 3.2.2, CDC, Atlanta, GA, USA) before the survey. First, individuals from 93 households which had been sampled in two of the villages by the International Food Policy Research Institute (IFPRI) and the University of Hohenheim, Germany, during household surveys in 2001 and 2003 (18) were re-interviewed. (Data from these households will be linked to previous surveys in future analysis.) Second, to ensure an adequate sample of people with HIV infection so that the differences between the HIV-affected and the unaffected households could be detected, 28 members from the three community AIDS support groups, identified during our qualitative study in 2006, were recruited. A positive HIV test is a requirement for membership in these groups (13). Third, additional households from these villages and four additional randomly-selected villages were randomly selected by surveying the 4th to 8th household from the village censuses provided by the chairman of each village. The sampling interval was based on the number of households from each village needed to yield an equal proportion of households from each village in the final sample. In total, 125 households were randomly selected using this method. There were no statistically significant differences in the demographic characteristics of households previously sampled by the IFPRI and those randomly selected for this study.

### Data collection

The survey instrument, which was developed by the authors and drawn from the previous IFPRI surveys conducted in the study area, was pre-tested in households near the study site, but not in study communities, in June 2007 and was revised for clarity before interviewing the study participants in July and August 2007. The questionnaire and consent forms were translated from English to Lusoga, the local language, and then back-translated from Lusoga to English to ensure accuracy. Two local research assistants trained in research methodology conducted interviews in Lusoga in the homes of the participants.

There were two components for the survey. First, the head of household, or another adult if the head of household was unavailable, was asked about household characteristics, including the number of residents, socioeconomic status, agricultural productivity, and labour allocation, and a global positioning system (GPS) location for the household was taken. Household heads were also asked about village land-rights and inheritance. The second part of the survey was an individual survey of all adults aged 18 years and older, including persons with HIV/AIDS, who were living in the household and were available for an interview. This survey asked about daily activities, and knowledge on and beliefs about HIV/AIDS, diet, and personal health status. Height and weight of each participant were also measured if the participant consented to anthropometry. (In total, 95.3% of the participants agreed to be measured.) When possible, individual interviews were conducted with all other adults in the household immediately after completing the interview with the head of household. If any adult member in the household was not available at that time, another time for the interview was scheduled. If two follow-up interview appointments were unsuccessful, the individual was deemed to be not interested in participation, and no further contact was initiated. The participants were interviewed privately so that their answers would not be influenced by the presence of other family members.

### Ethical considerations

The research ethics committees of the George Mason University (Fairfax, Virginia, USA) and the Uganda National Council for Science and Technology (Kampala) approved the research protocol. The community leaders and community groups were consulted before data collection was begun; they gave their approval for the research project to be conducted. Before starting household interviews, a statement that explained the purpose of the research, the risks and benefits of participation to individuals and communities, and the methods taken to ensure confidentiality of responses was read to all adults in the household. All individual research participants then provided written consent. No inducement or compensation was offered to participating individuals or households.

Although the aim of the study was to investigate the impact of HIV/AIDS on households, the participants were not tested for HIV, and no medical evaluation was conducted. The participants were asked whether they had been tested for HIV and, if they had been tested, were asked if they were willing to share the results with the interviewer or would prefer not to share the results of the test. All the participants were informed about free HIV testing and counselling facilities available in locations near the district.

### Statistical methods

Statistical analysis was conducted using the SPSS software (version 16.0) (SPSS Inc., Chicago, IL, USA). Comparisons between groups were made using chi-square tests, and the significance level was set at α=0.05.

## RESULTS

### Study population

In all, 322 (24.2%) of 1,333 adult residents from 246 participating households were interviewed. The most common reason given for not being able to interview a household member was that the household member was out of town (for example, the grown child of the head of household was attending a school in another town or was working in the city). Eight adults from the participating households were too ill to be interviewed.

### Classification of HIV

Based on the HIV status, the study population was divided into three groups: HIV-infected, HIV-affected, and HIV-unaffected. The HIV-infected group included 56 individuals (17.4% of the participants) who stated that they had tested positive for HIV infection. This is higher than the national prevalence rate of 6.7% because of oversampling from the AIDS support group (4). The HIV-affected group included 120 individuals (37.3%) who did not report being HIV-infected but reported having a household member with HIV infection. The HIV-unaffected group included 146 individuals (45.3%) who did neither report being HIV-infected nor currently having a household member with HIV infection. Table 1 shows the sociodemographic and HIV knowledge-related characteristics of these three HIV groups. There were no significant differences in the HIV status by sex, age, or years of education.

**Table 1.** Sociodemographic and HIV knowledge-related characteristics by HIV status$

Characteristics	HIV-infected (n=56)	HIV-affected (n=120)	HIV-unaffected (n=146)	p value for difference between HIV groups
Sex				
Female	42 (75.0)	76 (63.3)	87 (59.6)	0.125
Male	14 (25.0)	44 (36.7)	59 (40.4)	
Age (years)				
Mean age (standard deviation)	42.6 (10.8†)	41.0 (16.1†)	39.6 (1.3†)	0.445
Education				
0 year	15 (27.8)	26 (21.8)	33 (23.1)	0.646
Primary [(1–7 year(s)]	30 (55.6)	70 (58.8)	75 (51.4)	
Secondary (8+ years)	9 (16.7)	23 (19.3)	35 (24.5)	
Body mass index (kg/m^2^)				
Mean BMI (standard deviation)	21.0 (3.2†)	21.9 (3.6†)	22.5 (4.0†)	0.042*
% overweight (BMI ≥25.0)	5 (9.3)	24 (20.0)	20 (18.8)	
% underweight (BMI <18.5)	10 (18.5)	22 (18.3)	12 (9.0)	0.184
Familiarity with HIV/AIDS				
I know about HIV/AIDS	56 (100)	116 (96.7)	137 (93.8)	0.183
HIV testing and status				
I know people who have been tested	53 (94.6)	97 (80.8)	97 (66.4)	<0.001*
I know where to be tested for HIV	54 (96.4)	105 (87.5)	107 (73.3)	0.001*
I have been tested for HIV	56 (100)	59 (49.2)	69 (47.3)	<0.001*
Antiretroviral drug therapy				
I know someone taking ARVs	52 (92.9)	68 (56.7)	63 (43.2)	<0.001*
HIV transmission modes (free-response answer)				
Spread by sex	55 (98.2)	110 (91.7)	126 (86.3)	0.031*
HIV-prevention methods				
HIV is prevented by being faithful	53 (94.6)	110 (91.7)	123 (84.2)	0.111
HIV is prevented by abstaining from sexual intercourse	49 (87.5)	109 (90.8)	127 (87.0)	0.472
HIV is prevented by using a condom	52 (92.9)	94 (78.3)	107 (73.3)	0.048*

*p<0.05;

$HIV-affected: HIV-negative individuals with an HIV-infected household member;

HIV-unaffected: Households with no HIV-infected members;

†Standard deviation;

AIDS=Acquired immunodeficiency syndrome;

ARV=Antiretroviral;

BMI=Body mass index;

HIV=Human immunodeficiency virus;

Figures in parentheses indicate column percentages unless otherwise indicated

Of the 56 HIV-infected participants, 27 (48.2%) were recruited from three local AIDS support groups, 20 (35.7%) had previously participated in the IFPRI survey, and nine (16.1%) were from the households randomly selected from the community for inclusion. The participants recruited from the AIDS support groups were more likely to be aged over 40 years (p<0.001) and to be female (p=0.021) but the recruiting groups had statistically similar results for other demographic and health questions. (The age groups for ‘younger’ and ‘older’ adults are based on the median age of the participants.) The 56 HIV-infected participants reported significantly higher rates of diarrhoea in the past month (21.4% vs 11.3%, p=0.028) than the participants who did not state that they had HIV infection; there were no differences in the rates of tuberculosis (5.4% vs 2.3%, p=0.458), pneumonia (7.1% vs 9.8%, p=0.568), malaria (73.2% vs 71.1%, p=0.776), or fever (46.4% vs 47.0%, p=0.809). Only three of the HIV-infected participants were too ill to get out of bed for their interview.

### Knowledge on HIV

The study population was highly knowledgeable about HIV/AIDS. In all, 96.0% of the participants reported having knowledge on HIV/AIDS, 74.2% reported knowing someone with HIV, and 80.4% reported knowing someone who had died of AIDS. Also, 52.8% reported that, at some point (either currently or in the past), a member of their household had HIV/AIDS. When asked a free-response question about how HIV is transmitted, 90.4% correctly reported that it could be spread through sexual intercourse, 16.1% reported that it could be spread through blood, and 9.6% reported that it could be spread through needles. Most participants agreed that HIV could be prevented by being faithful (88.8%), abstaining from sexual intercourse (88.5%), and using a condom (78.6%). Only 14.6% of the participants thought that male circumcision could prevent HIV transmission. In total, 80.7% of the participants agreed that a person with HIV infection could look healthy. Females and males had similar levels of knowledge on HIV, as did younger adults and older adults.

The large proportion of the participants knew where to be tested for HIV (82.6 %) and knew people who had been tested (76.9%). In total, 57.1% of the participants reported that they had been tested for HIV. Of those who were tested, 96.2% had received the results of the test. Older adults were more likely to know where to be tested for HIV than younger adults (88.0% vs 76.7%, p=0.026) but the proportion of younger and older adults tested was not different (56.0% vs 58.2%, p=0.749). The HIV-infected participants were more likely to know where to be tested (p<0.001) and to have been tested (p<0.001). More than half (56.8%) of the participants reported knowing someone taking ARVs but only 22 participants reported taking ARV drugs, which is 39.3% of the people who tested positive for HIV and 6.9% of the sample population. This is less than the 56% rate of ARV-use reported for Uganda as a whole (4). Although ARVs are provided free of charge at the government clinics, ancillary costs for transportation, health facility user-fees, lost wages, and other financial burdens have been shown to reduce ARV adherence in Uganda (19), and the government health system has trouble keeping up with demand for ARVs (20).

### HIV and work

The survey included specific questions that investigated beliefs and behaviours relating to HIV status and reduction of working hours. Of the 322 participants in the survey, 299 (92.9%) reported that a person with HIV infection should work less to save energy, and 187 (58.1%) reported knowing people with an HIV diagnosis who had reduced their work-hours.

To further assess the work-practices, all the participants were asked several questions about the hours of time they spend in a typical day engaged in specific activities. For analysis, these activities were divided into three categories: (a) work outside the home, such as work in the family farm, work in the kitchen-garden (a vegetable plot near the home), or as paid labour; (b) work in the home, such as cleaning the house and yard, washing clothes, and cooking; and (c) time spent caring for children, elders, and sick family members. The respondents were asked to indicate how many hours or minutes during a typical day they participated in each activity. The respondents who reported working ‘all day’ or ‘all the time’ for any category were assigned to the highest work-hour group (Table 2).

**Table 2.** Work-hours by HIV status

Work-belief or practice	HIV-infected (n=56)	HIV-affected (n=120)	HIV-unaffected (n=146)	p value for HIV-infected vs HIV-affected	p value for HIV-infected vs all others	p value for HIV-affected vs HIV-unaffected
Proportion who believed that a person with HIV infection should work less to save energy	54 (96.4)	112 (93.3)	134 (91.8)	0.711	0.556	0.682
Proportion who believed that a person with HIV infection should rest more to save energy	56 (100)	114 (95.0)	129 (88.4)	0.235	0.074	0.080
Proportion who knew someone with HIV infection who has worked less to save energy	49 (87.5)	69 (57.5)	69 (47.3)	<0.001*	<0.001*	0.009*
Work-hours outside the home (in the farm, in kitchen-garden, or for pay)				
0	6 (10.7)	8 (6.7)	5 (3.4)			
>0–4	18 (32.1)	44 (36.7)	58 (39.7)	0.793	0.370	0.612
>4–7	15 (26.8)	33 (27.5)	37 (25.3)
>7	17 (30.4)	35 (29.2)	46 (31.5)			
Work-hours inside the home (cleaning, washing, cooking)				
0	13 (23.2)	28 (23.3)	40 (27.4)			
>0–3	13 (23.2)	21 (17.5)	31 (21.2)	0.836	0.890	0.379
>3–6	18 (32.1)	43 (35.8)	38 (26.0)
>6	12 (21.4)	28 (23.3)	37 (25.3)			
Care-giving hours (caring for children, the elderly, or the sick)					
0	18 (32.0)	38 (31.7)	53 (36.3)	0.985	0.719	0.327
>0–2	21 (37.5)	44 (36.7)	41 (28.1)
>2	17 (30.4)	38 (31.7)	52 (35.6)

HIV=Human immunodeficiency virus;

*p<0.05;

Figures in parentheses indicate column percentages unless otherwise indicated

Women reported working more hours than men (p<0.001), and younger adults reported working more hours than older adults (p<0.001). There were no significant differences in work-hours reported by HIV status when analyzing working time as either a continuous or a categorical variable (Table 2). Even those who reported illness in the past month did not report significantly fewer hours, except for participants who had been diagnosed in the past month with pneumonia (p=0.028) or had tuberculosis (p=0.011).

There might be some HIV-positive individuals who were not tested and diagnosed and were consequently misclassified as HIV-negative. The similarity in work-hours, therefore, could be due to misclassification of undiagnosed individuals with HIV who had illnesses that forced them to work fewer hours than healthier adults, a bias that would cause an artificially-low difference in the reported work-hour estimates between the groups. The results could also be the result of the sample population under-representing the healthiest or the sickest members of the community, which may have led to the failure to detect potentially real differences in work-hours for HIV-infected and other participants. However, given that our study participants expected persons who knew that they had HIV to work less and given that those whose status was unknown would not voluntarily reduce their work-hours, misclassification bias should not be a major concern when examining work-habits. Therefore, these results seem to indicate that although it is commonly believed that if it is possible to work fewer hours a person who has tested positive for HIV should rest more often, the reality is that few households can afford to allow a healthy person with HIV to work less.

### HIV and food

Of those who participated in the study, 91.6% believed that a person with HIV/AIDS should eat special foods. The HIV-infected participants identified a greater number of special foods (p<0.001) than the other population groups, especially for fruits (p<0.001) and vegetables (p=0.006) (Table 3). There were no differences in the types of ‘special foods’ or the number of ‘special foods’ reported by sex- or age-group.

**Table 3.** Food-related beliefs and food consumption in the past day by HIV status

Food belief or practice	HIV-infected (n=56)	HIV-affected (n=120)	HIV-unaffected (n=146)	p value for HIV-infected vs HIV-affected	p value for HIV-infected vs all others	p value for HIV-affected vs HIV-unaffected
Proportion who believed that a person with HIV infection should eat special foods	54 (96.4)	116 (96.7)	125 (85.6)	0.823	0.357	0.007*
Types of special foods for HIV/AIDS identified						
Any staple grain†	51 (91.1)	99 (82.5)	117 (80.1)	0.136	0.075	0.624
Any plant-based protein§	24 (42.9)	57 (47.5)	54 (37.0)	0.565	0.876	0.084
Any animal-protein‡	29 (51.8)	78 (65.0)	90 (61.6)	0.094	0.112	0.572
Any vegetable	33 (58.9)	44 (36.7)	44 (30.1)	0.006*	<0.001*	0.260
Any fruit	21 (37.5)	16 (13.3)	21 (14.4)	<0.001*	<0.001*	0.805
Number of special foods for HIV/AIDS identified						
Mean (standard deviation)	6.5 (2.0$)	5.2 (1.8$)	5.3 (2.0$)	0.001*	<0.001*	0.589
Types of food eaten in the past 24 hours						
Any staple grain	55 (98.2)	120 (100)	143 (97.9)	0.142	0.686	0.114
Any plant-based protein	31 (55.4)	72 (60.0)	85 (58.2)	0.560	0.613	0.769
Any animal-protein	20 (35.7)	39 (32.5)	54 (37.0)	0.674	0.915	0.445
Any vegetable	22 (39.3)	26 (21.7)	29 (19.9)	0.015*	0.003*	0.718
Any fruit	14 (25.0)	11 (9.2)	25 (17.1)	0.005*	0.031*	0.059
Number of different foods eaten in the past 24 hours						
Mean (standard deviation)	5.2 (1.8$)	4.3 (1.4$)	4.5 (1.2$)	0.001*	0.002*	0.379

*p<0.05;

†Such as sweet potato, cassava, maize, millet, mtoke/banana, rice, bread, yams, sorghum, or potatoes;

§Such as beans, peanuts, cowpeas, or soy-products;

‡Such as fish, meat, chicken, or eggs;

$Standard deviation;

AIDS=Acquired immunodeficiency syndrome;

HIV=Human immunodeficiency virus;

Figures in parentheses indicate column percentages unless otherwise indicated

People with HIV infection reported eating more special foods identified in the previous paragraph compared to other participants (Table 3), especially fruits (p=0.020) and vegetables (p=0.012). People with HIV also reported eating different varieties of foods than uninfected members of the community, which indicates a conviction that good nutrition is an essential component of maintaining health when infected with HIV. Females and males reported similar dietary habits. Younger adults reported eating more animal-protein (p=0.004) than older adults but there were no differences for the other food-groups.

## DISCUSSION

The study participants were highly knowledgeable about many aspects of HIV/AIDS, including transmission mechanisms, prevention methods, voluntary counselling and testing (VCT) sites, and treatment options. This is consistent with a previous evaluation of HIV/AIDS knowledge in Uganda that found a high level of knowledge on health and a remarkably high rate of personal connections with people known to have HIV/AIDS compared to other African countries (21). Most participants—whether they were HIV-infected, HIV-affected, or HIV-unaffected—expressed beliefs that persons diagnosed with HIV infection should reduce work-hours to conserve energy and should eat special nutritious foods to maintain health.

The study participants indicated a belief that people with HIV infection should work less to save energy if they have the resources to do so. This was expressed by the large majority of the participants in both the quantitative and qualitative surveys that we conducted. The comments made by participants in the 2006 qualitative survey described in the Introduction section of this paper provide additional insights into these findings. Most noted a pattern of working in the morning and resting in the afternoon. One member of an AIDS support group said:

When a person is HIV-positive and is still strong, he should rest on return from the garden. This is what we normally do. Even if you go for gardening, you have to minimize work-hours.

A community leader said:

A person can work a bit, but if you increase your work the illness will get you down. So, normally a person will return home from the garden early, depending on the amount that has been accomplished.

A health official who agreed with these recommendations and observations stated:

For a person who is positive, we encourage him to work but not excessively. We want them to take care of themselves. They normally get to the garden at 10 in the morning and return at noon. Once men return home they take a bath and rest and listen to radio but women start doing housework.

A farmer suggested alternative activities for those with HIV:

In the villages, the sick ones work from 8 to 10 am in the morning because they do not have energy. [In the afternoon,] the sick graze animals and attend their shops and buy produce at the market.

However, contrary to reports that reduced workload was not only encouraged but also frequently observed, the hours of work reported by the study participants were not significantly reduced for the HIV-infected participants compared to other participants. The community members who participated in our qualitative study provide explanations for this finding:

In most cases, people have to first work to get something to eat, so there is no way you can work less.

A person will work less depending on the type of family he has. If it is a big family, he has to work a lot to sustain them.

A person living with HIV/AIDS should not work but it depends. If he is the head of the house, he is forced to work.

An agriculture official raised concerns about overwork:

The patient usually does not work but it depends. If he is the head of the house, he is forced to work. In a week, he will farm for five days, but if he strains too much the disease can bring him down.

Financial realities and household needs generally do not allow for a relatively-healthy person with HIV infection to significantly cut back on work to rest more. It is possible that people with HIV infection are working the same hours as others but with less intensity; however, even a reduced work-intensity might not meet the locally-valued goal of ‘saving energy’ to promote a longer, healthier life.

The study participants indicated a belief that highly-nutritious foods, such as fruits and vegetables, ought to be consumed by people with HIV infection, which is in agreement with the recommendations of the Ugandan Government (12, 22), local AIDS support groups, and previous studies on nutrition for persons with HIV/AIDS (23,24) that suggest the importance of a diet that includes a variety of staple carbohydrates, sugars, fats and oils, dietary fibre, proteins, and vitamin- and mineral-rich vegetables and fruits. Good nutritional status has been shown to be associated with higher health status in persons with HIV infection (24–27).

In this study, the HIV-infected participants recruited through the community AIDS support group were more likely to believe that a person with HIV infection should eat more ‘special foods’ than persons with HIV recruited through random sampling of households in the community who were not members of the support groups (p=0.031). Members of the AIDS support groups who participated in the qualitative survey also touted the benefits of these foods:

Special foods are eaten to give more energy to the patient.

Whenever I eat these special foods, I feel good and get some energy.

The HIV-infected participants in the study reported consuming more fruits and vegetables than did other members of their households. This may put a strain on family members who might have to eat less nutritious food to ensure that the person with HIV has access to these nutrients. One sign of potential stress in our study population is that both HIV-infected persons and their household members were more likely than participants from unaffected households to be underweight. A related concern is that previous studies have found that a low body mass index (BMI) is associated with reduced ability to work (28,29), which might further compromise household food security. Furthermore, the relatively-high rate of overweight in our population when compared with other studies from Uganda (30,31) supports a previous observation that food insecurity is associated with a higher prevalence of overweight (32).

In the study area, members of the AIDS support groups are eligible to receive free food supplements from NGOs, such as The AIDS Support Organization (TASO), an NGO that provides medical care, social support, and education and counselling on HIV/AIDS to its clients. The typical monthly foods distributed to the HIV-infected support group members in this community include staple foods, such as about 10 kilograms of corn-meal, 12 kilograms of soy-flour, 5 kilograms of cowpeas and beans, and 3 litres of cooking-oil. This package is intended as a supplement only and does not include any foods identified as ‘special’ nutritious foods. Since fruits and vegetables are not part of the food-package, household resources must be used for acquiring these foods. While it is possible that the free supplemental food allows households to divert money they would have spent on staple foods to purchase fruits and vegetables, many study participants had difficulty acquiring these foods. For example, one woman stated:

It is true about special foods but they just eat what is available.

And a community leader said:

It is only when you have enough resources that you can eat special foods.

There are several limitations to this study. First, our recruitment of members of the HIV/AIDS support groups may have introduced selection bias in which the participants with HIV infection are not representative of all persons living with HIV in the study area. Second, since we did not test participants for HIV, misclassification bias may have occurred in our categorization of individual or household HIV status. However, since the specific aim of the study was to understand behavioural changes that occur in otherwise healthy people following an HIV diagnosis and since only persons who know they have HIV and had disclosed their status would be expected to make significant lifestyle changes, it is reasonable for the purposes of this study to classify as HIV-infected only those persons who chose to disclose their status to interviewers. Third, we may have encountered information bias because we did not require the participants to keep a food diary or maintain a log of work-hours, and the participants reported on their own health status. Previous studies of self-reports of health status have shown that this may lead to over- or under-estimation of health status (29).

In conclusion, the results of this study of HIV-related knowledge, beliefs, and practices in rural Uganda revealed that the study population had a high level of knowledge on HIV. While most participants believed that a person with HIV infection should work less to conserve energy, even if that person was relatively healthy, a significantly-reduced workload among those with HIV infection was not found. The HIV-infected participants, however, reported eating more fruits and vegetables than did other participants. Further research is needed to determine how ‘special foods’ and reduced labour might contribute to longer, healthier lives for people with HIV in Uganda.

More importantly, the findings of this survey highlight the large extent to which local knowledge and beliefs influence local health practices. The HIV/AIDS organizations working in the study communities in Uganda have developed assistance programmes that specifically address perceived local needs. While the findings of this study on diet and labour may not be generalizable to other regions in the world, the results do highlight the need for health educators and policy-makers in all communities to understand local expectations before developing and implementing health policies and programmes.

## ACKNOWLEDGEMENTS

Funding for this research was provided by a Leadership Enhancement Agricultural Program grant from the Norman E. Bourlag International Agricultural Science and Technology Fellow's Program, the International Food Policy Research Institute (IFPRI), the Robert Bosch Foundation, and George Mason University. The authors thank IFPRI-Kampala, the Uganda National Council of Science and Technology, and Mayuge District officers for their assistance. This work could not have been completed without the assistance of the local research assistants Claire Nakayega, Albert Mdhugumbya, and Maureen Ndahura, and the hospitality of the villages and households that participated in the survey.
